# IL-6 is associated to IGF-1Ec upregulation and Ec peptide secretion, from prostate tumors

**DOI:** 10.1186/s10020-018-0003-z

**Published:** 2018-03-15

**Authors:** A. Armakolas, A. Dimakakos, C. Loukogiannaki, N. Armakolas, A. Antonopoulos, C. Florou, P. Tsioli, E. Papageorgiou, T. P. Alexandrou, M. Stathaki, D. Spinos, D. Pektasides, E. Patsouris, M. Koutsilieris

**Affiliations:** 1 Physiology Laboratory, Medical School, National & Kapodistrian University of Athens, 115 27 Goudi-Athens, Greece; 2 Third orthopaedic clinic, KAT General Hospital, 145 61 Kifisia, Attiki Greece; 3 Oncology Section, Second Department of Internal Medicine, Hippokration Hospital, 115 27 Athens, Greece; 4 Department of Pathology, University of Athens, Medical School, 115 27 Athens, Greece

**Keywords:** Immune response, IGF-1Ec, Il-6, Il-6R, JAK2/STAT3 pathway, MSCs mobilisation

## Abstract

**Background:**

Ec peptide (PEc), resulting from the proteolytic cleavage of the IGF-1Ec isoform, is involved in prostate cancer progression and metastasis, whereas in muscle tissue, it is associated with the mobilization of satellite cells prior to repair. Our aim is to determine the physiological conditions associated to the IGF-1Ec upregulation and PEc secretion in prostate tumors, as well as, the effect of tumor PEc on tumor repair.

**Methods:**

IGF-1 (mature and isoforms) expression was examined by qRT-PCR, both in prostate cancer cells co-incubated with cells of the immune response (IR) and in tumors. PEc secretion was determined by Multiple Reaction Monitoring.

The effect of PEc, on mesenchymal stem cell (MSC) mobilization and repair, was examined using migration and invasion assays, FISH and immunohistochemistry (IHC). The JAK/STAT signaling pathway leading to the IGF1-Ec expression was examined by western blot analysis. Determination of the expression and localization of IL-6 and IGF-1Ec in prostate tumors was examined by qRT-PCR and by IHC.

**Results:**

We documented that IL-6 secreted by IR cells activates the JAK2 and STAT3 pathway through IL-6 receptor in cancer cells, leading to the IGF-1Ec upregulation and PEc secretion, as well as to the IL-6 expression and secretion. The resulting PEc, apart from its oncogenic role, also mobilizes MSCs towards the tumor, thus promoting tumor repair.

**Conclusions:**

IL-6 leads to the PEc secretion from prostate cancer cells. Apart from its oncogenic role, PEc is also involved in the mobilization of MSCs resulting in tumor repair.

**Electronic supplementary material:**

The online version of this article (10.1186/s10020-018-0003-z) contains supplementary material, which is available to authorized users.

## Background

The IGF-1 gene gives rise to three premature isoforms, all of which, after proteolytic cleavage, result in the mature IGF-1 (IGF-1) that consists of the peptide products of parts of exon 3 and 4. Under normal conditions, the major isoform produced is the IGF-1Ea (peptide products of exons 3, 4 and 6), whereas IGF-1Eb (peptide products of exons 3, 4 and 5) and IGF-1Ec (peptide products of exons 3, 4 and parts of exons 5 and 6) are expressed in almost negligible amounts. The Ec peptide (PEc or MGF) consists of the last 24 aa of the IGF-1Ec (Philippou et al., 2014). The results of the overexpression and silencing of PEc in prostate cancer cell lines in in vitro and in vivo models suggest that PEc is one of the key players in prostate cancer progression and metastasis by activating the ERK 1/2 pathway and therefore inducing cellular proliferation and Epithelial to Mesenchymal Transition (EMT), in prostate cancer cells. PEc seems to act in an autocrine and/or paracrine mode of action, through an unknown receptor**.** Other than the IGF-1R, Insulin Receptor, or any of the IGF-1/Insulin Receptor hybrids that are involved in the IGF-1 system (Armakolas et al., 2010; Armakolas et al., 2015). Consistent with our laboratory evidence**,** PEc expression in human prostate cancer biopsies was significantly associated to tumor grade and stage (Armakolas et al., 2015; Savvani et al., 2013).

Despite the involvement of PEc in cancer and its lack of expression in normal tissue**,** the physiological role of PEc**,** up to now**,** is closely interwoven with skeletal and cardiac muscle as part of their repair and regeneration process. PEc has been proposed to mobilize the satellite cells (myogenic stem cells) as a response to stimuli such as injury or exercise in the former case and myocardial infarction in the later (Kandalla et al., 2011; Matheny Jr. et al., 2010; Philippou et al., 2009; Stavropoulou et al., 2009; Mavrommatis et al., 2013; Hill et al., 2003; Hill & Goldspink, 2003).

There is, therefore, the possibility that prostate tumor damage may lead to PEc secretion and PEc itself may be associated with the tumor repair process, by inducing human mesenchymal stem cell (HMSC) mobilization, similarly to its effects in skeletal and cardiac muscle. The expression level of the IGF-1Ec isoform in normal prostate is negligible (Dai et al., 2010), as is the case in advanced prostate cancer cell lines when growing in in vitro condition (Armakolas et al., 2010; Armakolas et al., 2015). This indicates that the IGF-1Ec isoform that leads to the production of PEc is expressed only when required.

A previous study where subcutaneous tumors had been generated in SCID mice using PC-3 prostate cancer cells (advanced prostate cancer), resulted in the interesting observation that, 1 week after palpable tumor detection, IGF-1 Ec was moderately expressed (Armakolas et al., 2015).

It is now widely accepted, that human tumors are immunogenic (Blankenstein et al., 2012; Hernandez et al., 2016), therefore the immune response (IR) has an adverse effect on tumor growth. SCID mice do not possess T or B lymphocytes, the cells that are responsible for the adaptive IR but retain their innate immune response mechanism (IIR) intact (Bastide et al., 2002).

Our aim was to determine the in vivo physiological mechanism that leads to the IGF-1Ec upregulation and PEc secretion in prostate tumors. In addition, since PEc is also associated with MSC mobilization and repair in healthy tissue, we also examined the effects of tumor secreted PEc on HMSC mobilization and tumor repair.

## Methods

### Subjects

Human bone marrow was collected from three male healthy donors 32, 37, 29 years old with open femur fractures. Lymphocytes were isolated from whole blood from two 26 and 37 year old healthy males. A written informed consent (IC) was obtained by all subjects in any case. These IC as well as the entire study had been approved by the Institutional Ethics Committee. Animal studies have been approved by the Ministry of Rural Development and Food, General Directorate of Veterinary and all the experimental procedures conformed to the Declaration of Helsinki.

### Cell cultures

The PC-3 cells, an androgen resistant, p53-negative and Kirsten-Ras (K-Ras) mutated human prostate cancer cell line and the DU-145 human prostate cancer cell lines were obtained from American Type Cell Culture (ATCC, Manassas, VA, USA). Low passage (passage 10–15) PC-3 cells were maintained in Dulbecco’s modified Eagle’s medium (Cambrex, Walkersville, MD, USA) supplemented with 10% heat-inactivated foetal bovine serum (FBS) (Biochrom, Berlin, Germany) and 100 U/ml penicillin/streptomycin (Cambrex, Walkersville, MD, USA). Low passage DU-145 cells were maintained in Minimum essential media supplemented with FBS and 100 U/ml penicillin/streptomycin (all from GIBCO BRL, Invitrogen Corp, Carlsbad, CA, USA) at 37 °C in a humidified atmosphere of 5% CO2.

Subcutaneous injections in SCID mice.

Male SCID mice (7 weeks old) were obtained from Democretos Laboratory. Mouse handling and experimental procedures were approved by the Hellenic Ministry of Rural Development and Food, General Directorate of Veterinary. Animal handling and experimental procedures were obtained in the Experimental Surgery Laboratory of the Athens Medical School. Implantations were carried out as previously described (Armakolas et al., 2015). Briefly, a suspension of 1 X 107 cells in 200 μl in 1X PBS was injected subcutaneously in each mouse.

### Quantitative real-time PCR (qRT-PCR)

Total RNA was isolated using Trizol (Invitrogen, Carlsbad, CA, USA). Quantitative real-time PCR was performed in the Biorad IQ5 multicolor Real Time PCR detection system, as previously mentioned,). Each reaction was obtained in 25 ml using 12 ml SYBR green Supermix (Bio-Rad Laboratories Hercules, CA, USA), 0.5 mg/ml oligo dTs (Fermentas, Glen Burnie, MD, USA), 2 ml cDNA, and 0.3 mM primers. Each reaction was performed in triplicate and values were normalized to GAPDH. The primers used are summarized in Table 1. The PCR conditions were the same in all cases: 95 °C for 30 s 1 cycle, 94 °C 20 s, 60 °C 30 s 72 °C 30 s for 35 cycles and 72 °C for 5 min. The normalization of qRT-PCR experiments was carried out according to ΔΔct method using as a reference gene GAPDH and is the unscaled analysis from the software Biorad IQ-5 vs 2.0.

**Table 1:** Primers used in this study

Gene	Forward	Reverse
IGF-1Ec (PEc)	5′-TATCAGCCCCCATCTACCA-3′	5′-CTTGCGTTCTTCAAATGTACTTCCT-3′
IGF-1Ea	5′-GTATTGCGCACCCCTCAAG-3′	5′-CAAATGTACTTCCTTCTGGGTC-3′
IGF-1Eb	5′-GTATTGCGCACCCCTCAAG-3′	5′-CTACTTCCAATCTCCCTCCTCTG-3′
E- cadherin	5′-TGGAGGAATTCTTGCTTTGC-3′	5′-CGTACATGTCAGCCAGCTTC-3′
Vimentin	5′-GACAATGCGTCTCTGGCACGTCTT-3′	5′-TCCTCCGCCTCCTGCAGGTTCTT-3′
Mouse MIG-1	5′-TGAAGTCCGCTGTTCTTTTCC-3′	5′-GGGTTCCTCGAACTCCACACT-3′
Mouse IFNγ	5’-CTGCGGCCTAGCTCTGAGA-3′	5′-CAGCCAGAAACAGCCATGAG-3′
Human IFNγ	5′-CTAATTATTCGGTAACTGACTTGA-3′	5′-ACAGTTCAGCCATCACTTGGA-3′
Human NCAM	5′CATGGAATTAGAGGAGCAGGTCAC-3′	5′-CAGTGTACTGGATGCTCTTCAGG-3′
Mouse NCR1	5′-TGGCTCTTACAACGACTATGC-3′	5′-AGAAGAAGTAGGGTCGGTAGGTG-3′

### Lymphocyte isolation

Ficoll-Paque Plus (GE-Healthcare Bio-Sciences, Pittsburgh, PA 15264–3065 USA) was used according to manufacturers instructions. Briefly, one volume of blood in EDTA was mixed with one volume of PBS and was then gently poured on top of the Ficoll-Paque plus and centrifuged at 400 Χ g for 30 min at 20 °C. The middle lymphocyte layer was then transferred into a clean tube. The obtained cells were incubated in DMEM 10% FBS media. Each sample was analyzed for the presence of lymphocytes with flow cytometry on a FACS Calibur CyFlow ML Partecflow cytometer, using a mouse anti-human APC conjugated CD-45 antibody and a mouse anti-human FITC conjugated CD-3 antibody for T lymphocytes and CD-45 and a mouse anti-human FITC conjugated CD-20 antibody for B lymphocytes detection all from (Abcam, Cambridge, UK). The results obtained were analyzed using the ModFit software (Flowmax3.0 Software (1997–2007) Version 3.0 (b4)) (Additional file 1: Figure S1).

### Lymphocyte sensitization

PC-3 and DU-145 prostate cancer cells were treated for 30 min at 37 °C with 20 μg/ml Mitomycin C (Sigma Aldrich, St. Louis, MO, USA. These cells provided the monolayer upon which the Lymphocytes were to be sensitized. Approximately 2 × 107 lymphocytes were added in 3 ml of DMEM medium to each sensitizing culture flask (Kall et al., 1976).

### Human polymorphonuclear cell isolation

human polymorphonuclear cells were obtained from the blood of healthy donors after the depletion of lymphocytes using a combination gradient composed of equal volumes of Ficoll HistoPaque 1077 and Ficoll Histopaque 1119 (both from Sigma Aldrich, St. Louis, MO, USA). One volume of blood in EDTA was mixed with one volume of PBS and was then gently poured on top of the Ficoll-Paque plus and centrifuged at 400 Χ g for 30 min at 20 °C. The polomorphonuclear layer was then transferred into a clean tube. The obtained cells were incubated in DMEM 10% FBS media. Characterisation and counting of the cells was carried out by using a blood analyser (Sysmex XE 5000, Sysmex Sverige, Marios Gata 13, S-434 37 Kungsbacka, Japan) where it was determined that our cells were > 95% polymorphonuclears.

### Co-incubation protocol

Approximately 150–200,000 prostate cancer cells were co-incubated with 0.5 × 106 cells of the IIR or human lymphocytes for 24 and 48 h standard in vitro killing assay conditions (Hicks et al., 2006).

### Trypan blue exclusion assay

PC-3 and DU-145 cells were plated at a cell density of 12 × 103 cell/well in 24-well plates and grown with DMEM containing 10% FBS. These cells were co-incubated with cells of the IR. After 24 h and 48 h of seeding cells, the cells of the IR (lymphocytes or polymorphonuclears) were washed away and the cell number of the adhering prostate cancer cells was counted as previously described (Armakolas et al., 2010).

### Western blot analysis

Cell extracts were obtained, by lysis in RIPA buffer (50 mmol/L Tris-HCl, 150 mmol/L NaCl, (Sigma Aldrich, St. Louis, MO, USA) containing 0.55 Nonidet P-40, protease 1 mmol/L phenylmethylsulfonyl fluoride (PMSF) (Sigma Aldrich, St. Louis, MO, USA), 10 μg/mL aprotinin, 10 μg/mL leupeptin; (Sigma Aldrich, St. Louis, MO, USA) and phosphatase inhibitors (1 mmol/L sodium ortovanadate, 1 mmol/L NaF) (Sigma Aldrich, St. Louis, MO, USA) as previously described. Protein concentrations were determined by Bio-Rad Protein Assay (Bio-Rad Laboratories, Hercules, CA, USA). Equal amount of cell lysates (20 μg) were heated at 95 °C for 5 min, electrophoresed on 12% SDS–PAGE under denaturing conditions and transferred onto nitrocellulose membrane (Bio-Rad Laboratories, Hercules, CA, USA). The blots were blocked with TBS-T (20 mmol/L Tris–HCl [pH 7.6], 137 mmol/L NaCl and 0.1% Tween 20) containing 5% nonfat dried milk or 3% Bovine Serum Albumin (BSA) in the case of phospho antibodies, at room temperature for 1 h. The membranes were probed overnight with primary antibodies at 4 °C.

The primary antibodies used were: mouse monoclonal anti-human phospho STAT1–6 antibodies and rabbit polyclonal anti-human antibodies for total STAT1–6 (all at 1/1000 dilution, all from (Santa Cruz Biotecnology, CA, USA), and mouse monoclonal anti–human total JAK1–3 antibodies and rabbit monoclonal anti-human pJAK1–3 antibodies (all were used at 1/1000 dilution, (Abcam, Cambridge, UK) as an internal control we used the GAPDH. Detection was carried out by using an HRP goat anti-rabbit IgG or a goat anti-mouse IgG (both used at 1/2000 dilution, (Santa Cruz Biotecnology, CA, USA) and the Supersignal West Pico Chemiluminescent substrate kit (Thermo Scientific, Waltham, MA, USA).

Multiple reaction monitoring (MRM).

This analysis was performed as a fee for service in the centre de Recherche Protéomique, CHUL, G1V 4G2, Quebec, (Quebec) Canada. Briefly media from prostate cancer cells before and after the co-incubation with cells of the IR were collected and treated with dithiothreitol (DTT) and iodoacetamide and they were then digested overnight with trypsin. The samples were applied on a stage tip before mass spectrometry analysis.

Samples have been solubilised into 50 μL of 0.1% formic acid containing 5 fmol/μL of a standard peptide (ASSILAT). For each sample, 2 μL were injected into a 5500 Qtrap (AbSciex, Framingham, MA, USA). Peptides were eluted over an 18 min acetonitrile gradient and three peptides were monitored: YQPPSTNK and GSTFEER (PEc fragments) and ASSILAT (normalization). For each peptide, at least five transitions were monitored to improve the signal specificity.

### Human mesenchymal stem cell (HMSC) isolation

The whole procedure was carried out as previously described (Soleimani & Nadri, 2009; Armakolas et al., 2016). Briefly the obtained bone marrow was filtered through a 70-mm filtered mesh and the obtained cells were cultured at a density of 25 X 106 cells in DMEM, 20% FBS. The cells were incubated in a standard tissue culture incubator (37 °C and 5% CO2) the media was changed initially at 3 h and then every 8 h for the rest 72 h. The adherent cells were then trypsinised for 2 min at 25 °C and the obtained cells were examined for E-cadherin (epithelial marker) and Vimentin (mesenchymal marker) expression by immunofluorescence (Additional file 2: Figure S2).

### Immunofluorescence staining

Cultured cells on chamber slides were stained by an indirect immunofluorescence method. Cells were rinsed in PBS and fixed with ice cold 80% methanol for 10 min at room temperature. They were permeabilized with PBS plus 0.5% Triton X-100 (Sigma Aldrich, St. Louis, MO, USA) for 10 min. They were then incubated with primary antibodies overnight at 4 °C: rabbit anti-E-cadherin (1:100) (Abcam, Cambridge, UK) or mouse anti-Vimentin (1:100) (Abcam, Cambridge, UK) in PBS. After 3 washes with PBS, 5 min at room temperature, cells were incubated for 30 min with goat anti-rabbit IgG conjugated to the fluorescent Alexa 488 dye (1:2000) (Abcam, Cambridge, UK) or with goat anti-mouse IgG conjugated to the fluorescent Alexa 555 dye (1:2000) (Abcam, Cambridge, UK) in PBS. After 3 washes, samples were stained with DAPI (1 μg/ml) for viewing with microscope (Olympus BX40, Tokyo, Japan) (Armakolas et al., 2015).

### Migration/invasion assay

The effect of the secreted Ec peptide on HMSC migration and invasion was analyzed using Cultrex cell invasion assay (Trevigen, Gaithersburg, MD, USA) according to the manufacturer’s instructions. Briefly, for the invasion assay, the membrane in the upper chamber of 96-well plate was coated with 0.5 × basement membrane matrix/Matrigel. For the migration assay, the membrane was left uncoated. Human Mesenchymal Stem Cells were starved in the serum-free medium for 8 h prior to the assay, then seeded at the upper chamber at a density 5 × 104/well. Media from PC3, DU-145 and PC-3 PEcKD cells under the effect of the IR (48 h) was introduced at the bottom chamber. After 16 h, the cells on the lower surface were dissociated and counted using an inverted microscope prior to quantify the relative cell migration/invasion.

Fluorescence IN SITU Hybridisation (FISH) in Paraffin-Embedded Tissues Sections.

FISH was carried out according to the manufacturers instructions (Cambio Ltd., The Irwin Centre, Scotland Road, Dry Drayton, Cambridge, UK). Briefly slides were deparafinized in xylem and dehydrated in a sequential series of ethanol baths (100%, 96%, 80%, 70%). They were incubated in Pepsin solution (ZytoVision GmbH Fischkai 1D-27572 Bremerhaven, Germany) Quench the pepsin in Glycine solution. Post-fix in paraformaldehyde solution for 2 min. and apply 10 μl paint mix to the centre of the slide (Cambio Starfish Pan Centromeric Chromosome Paint 1697-MF-01, Scotland Road, Dry Drayton, Cambridge, UK). The slides were then denatured at 60 °C for 10 min and hybridised at 37 °C overnight. After washing in formamide and detergent wash solution the slides were incubated with DAPI (Life Technologies, Carlsbad, CA, 92008, USA) for 5 min at room temperature and they were then covered with Fluorescence Mounting Media (DAKO, 2966 Industrial Row Troy, Michigan 48084, USA). Visualization took place using a microscope (Olympus BX40, Tokyo, Japan) and the software used was the Case Data Manager 5.5 (Applied Spectral Imaging, Inc. 5315 Avenida Encinas, Suite 150 Carlsbad, CA 92008, USA).

### IL-6 blocking

IL-6 was blocked with a mouse anti-human IL-6 antibody (1/100), (Thermo Fisher Scientific, Waltham, MA, USA) and IL-6R was blocked with 100 nM Tocilizumab (Acterman, Roche Ltd. 2015 F. Hoffmann-La, Basel, Switcherland) an anti-IL-6 receptor antibody, in two different occasions. Briefly each antibody was added in cell culture just after the incubation of prostate cancer cells with human cells of the innate or adaptive IR or 1 h before the administration of IL-6. Cells were then collected and analyzed for the IGF-1Ec expression, for IL-6 expression and for JAK2 and STAT-3 activation.

### Immunohistochemistry

Formaldehyde-fixed tumors were paraffin wax embedded. Microtome sections of 3 μm were allowed to adhere to glass slides, dried at 37oC overnight, de-waxed in xylene and rehydrated in serial dilutions of ethanol. Antigen retrieval was obtained by heating the slides in a steamer in Envision Flex Retrieval Solution pH 9 (DAKO 50X in ddH2O), (DAKO, Glostrup, Denmark) covered with aluminum foil for 30 min. Serial sections of the tumors were then incubated with either anti-human IL-6 (1/20 in PBS) (Santa Cruz Biotecnology, CA, USA) or the polyclonal rabbit anti-PEc antibodies (1/5000 in PBS) or the rat anti-mouse CD-45 (1/20) (Thermo Fisher Scientific, Waltham, MA, USA) overnight at 4oC. The samples were then incubated with Biotinylated Link Universal (LSAB+ Kit, DAKO) (DAKO, Glostrup, Denmark) at RT in a humidified chamber for 30 min. Tissue sections were then visualized under light microscopy (Nikon Eclipse 80i; Nikon, Tokyo, Japan), while negative and positive control staining procedures were also included in all immunohistochemical analyses. Samples were photographed using a digital camera (Nikon DS-2 MW; Nikon, Tokyo, Japan). Image analysis was performed in six random fields from each slide using the Image Pro Plus 5.1 software (Media Cybernetics, Bethesda, MD, USA). IGF-1Ec (brown staining) average intensity levels, measured using arbitrary units on a linear scale from 0 (black) to 255 (white), and the average percentage of the extent of brown staining are combined in the following equation: PEc or IL-6 expression = 255 – average intensity levels of brown staining × average percentage of extent of brown staining. The mean intensity and extent of levels of brown staining for the six representative optical fields was estimated in each case and compared.

### Statistical analysis

Comparisons of IGF-1 isoform expression and mature IGF-1 expression between groups, was obtained and one-way Anova test Samples remained significant after Bonfferoni correction was applied whenever ANOVA test used and each pair of variables was also compared using the 2-tailed equal variance Student’s t test whereas the normal distribution was tested with the Kolmogorov-Smirnov (K-S) test (SPSS v. 11 statistical package, SPSS Inc. Headquarters, Chicago, USA). Statistical significance was set at *p* values less than 0.05, error bars refers to standard deviations (s.d), n = the number of experimental repeats.

## Results

**Fig. 1:**
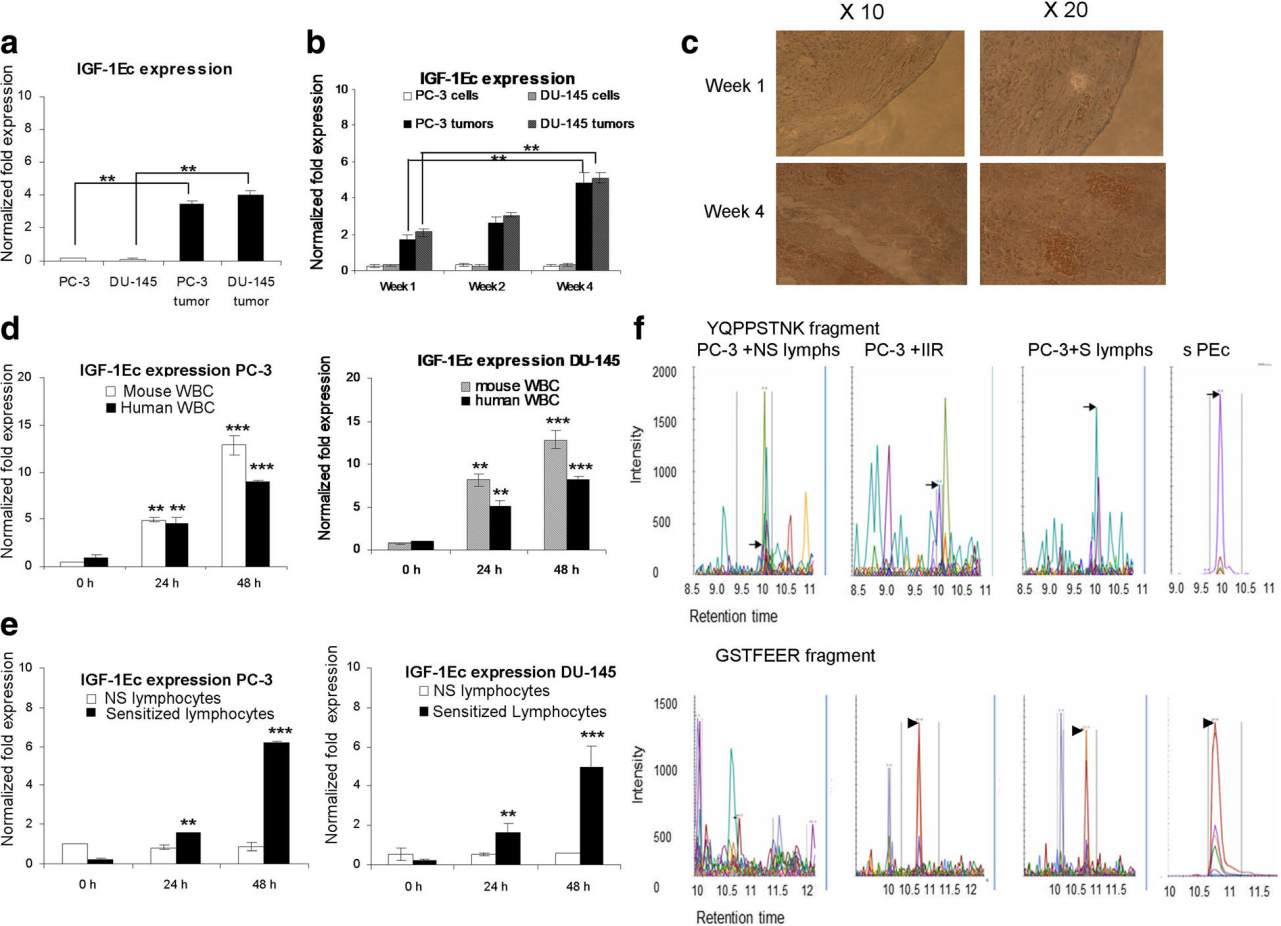
PEc expression is induced in prostate cancer cells as a response to the immune system attack. **a**: Analysis of IGF-Ec expression in PC-3 and DU-145 cell lines and their corresponding tumors in SCID mice, by qRT-PCR. The PC-3 and the DU-145 tumors produced significantly higher IGF-1Ec expression when compared to their corresponding cell lines. **b**: Analysis of IGF-1Ec expression in PC-3 and DU-145 tumors (qRT-PCR) at different time intervals. In both cases IGF-1Ec expression is increased as tumor progresses. **c**: Detection of mouse CD-45 positive cells in human tumors extracted from SCID mice (IHC). A significant increase of CD-45 cells was observed as tumor progresses. **d**: Effect of the mouse and human cells of the IIR in the IGF-1Ec expression levels in PC-3 and DU-145 cells. In both cases human and mouse IR seems to be associated with significant IGF-1Ec upregulation in prostate cancer cells at 24 and 48 h. **e**: Co-incubation of PC-3and DU-145 cells with human sensitized lymphocytes indicated a significant increase of the IGF-1Ec expression at 48 h compared to the prostate cancer cells treated with non-sensitized lymphocytes. **f**: Multiple Reaction Monitoring (MRM) analysis of protein in the media obtained from co-cultures of PC-3 cells with human lymphocytes or with cells of the IIR. Both PEc specific digest products (**YQPPSTNK** and **GSTFEER**), were detected into the media of PC-3 cells after co incubation with either the human cells of the IIR (sample 2) or with sensitized lymphocytes (sample 3) (**: *p* < 0.005, ***:*p* < 0.0005)

PC-3 and DU-145 cells as well as their tumors respectively, were quantitatively examined for the expression of the IGF-1Ec isoform. It was determined that the tumors arising from both cell lines presented a statistically significant IGF-1Ec elevation compared to the levels of their corresponding cell lines (*p* < 0.0001 in both cases, students t test, *n* = 3) (Fig. 1a). The IGF-1Ec expression levels in the subcutaneous tumors were also examined at different time intervals at 1, 2 and 4 weeks after palpable detection (n = 3 tumors per different time interval). IGF-1Ec expression was significantly increased as tumors progressed for both PC-3 and DU145 tumors (week 2 vs week 4 *p* = 0.005 and *p* = 0.0046 respectively, week 1 vs week 4 *p* = 0,00038 and *p* = 0,00033 respectively one-way Anova test, n = 3) (Fig. 1b). The activation of the IIR generated by the prostate cancer establishment in vivo was examined by assessing the expression of mouse MIG-1, an angiostatic cytokine induced by IFNγ, that can be secreted by the natural killer cells of SCID mice and plays physiologically important roles in promoting innate and adaptive IR (Haabeth et al., 2011). MIG-1 was elevated in white blood cells isolated from blood extracted from SCID mice after the establishment of subcutaneous tumors (Additional file 3: Figure S3 A) indicating a tumor associated activation of the IR. The attack of the SCID mice IR on prostate tumors was determined by IHC. An increased amount of mouse CD-45 (pan-leucocyte marker) positive cells, was observed to co-localize to the tumors (periphery and inside), that increased as the tumors progressed (Fig. 1c).

### In vitro innate immune response (IIR) activation by prostate cancer cells

Blood was extracted from SCID mice and after red cell lysis the rest of the cells (neutrophils, eosinophils, basophils and macrophages), were incubated with PC-3 or DU-145 cells for 0, 6 and 12 h. Mouse MIG-1 levels were significantly induced after 6 and 12 h co-incubation with prostate cancer cells and SCID mouse blood cells (Additional file 3: Figure S3 B).

### In vitro effect of the immune response (IR) in prostate cancer cells

Cells of the IIR or sensitized lymphocytes were co-incubated with either PC-3 or DU-145 cells and cellular viability of the prostate cancer cells was measured at 24 h. It was found that prostate cancer cell numbers were statistically significant decreased (*p* < 0.009 for every case, students t test, *n* = 3, Additional file 3: Figure S3 C), indicating the in vitro activation of the innate and adaptive IR.

#### Effect of IIR on IGF-1Ec expression

The effect of the mouse and human IIR was also examined on IGF-1Ec expression. SCID mouse blood cells were incubated with PC-3 and DU145 cells for 0, 12, 24 h. A statistically significant increase in the IGF-1Ec expression was determined at 24 h (Fig. 1d, e). Similar results were obtained after co-culturing freshly isolated human white blood cells with PC-3 and DU-145 cells (*p* = 0.0048 and *p* = 0,0034 respectively, students t test, *n* = 3).

### Adaptive IR

Prior to determining the effect of the human adaptive immune respοnse to cancer produced PEc, freshly isolated human lymphocytes, were sensitised to PC-3 and DU-145 cells and were then co-cultured with PC-3 and DU-145 respectively. The intracellular expression of the IGF-1Ec isoform presented a 6 fold increase after 48 h incubation of PC-3 cells with human sensitized lymphocytes (SL), compared to the PC-3 cells treated with non-sensitized lymphocytes (*p* < 0.001, students t test *n* = 3, triplicate) Similar were the results for DU-145 cells (*p* < 0.001 students t test, *n* = 3, triplicate), (Fig. 1d, e). Determination of the secreted PEc in PC-3 cells was obtained with MRM by examining the extracellular content of the PC-3 cells after incubation with SL for the presence of PEc. It was confirmed that PC-3 cells secreted PEc as a response of the immune system attack (Fig. 1f).

### Effect of the human IR on IGF-1 isoforms

The IGF-1 isoforms expression ratio under the effect of either the human IIR was also examined. It was found that in both prostate cancer cell lines, the expression of the IGF-1Ea isoform was not significantly affected by the innate or adaptive IR (results not shown). Similarly to the IGF-1Ec, IGF-1Eb isoform presented a significant increase at 24 and 48 h compared to the untreated cell lines (in PC-3 cells *p* = 0.0001 and in DU-145 *p* = 0,00009), students t test, *n* = 3) (Additional file 4: Figure S4 A-D). This increase of the two isoforms was not reflected on the mature IGF-1 levels, where no statistically significant increase was observed (results not shown).

### Effect of PEc on the HMSC mobilization

**Fig. 2:**
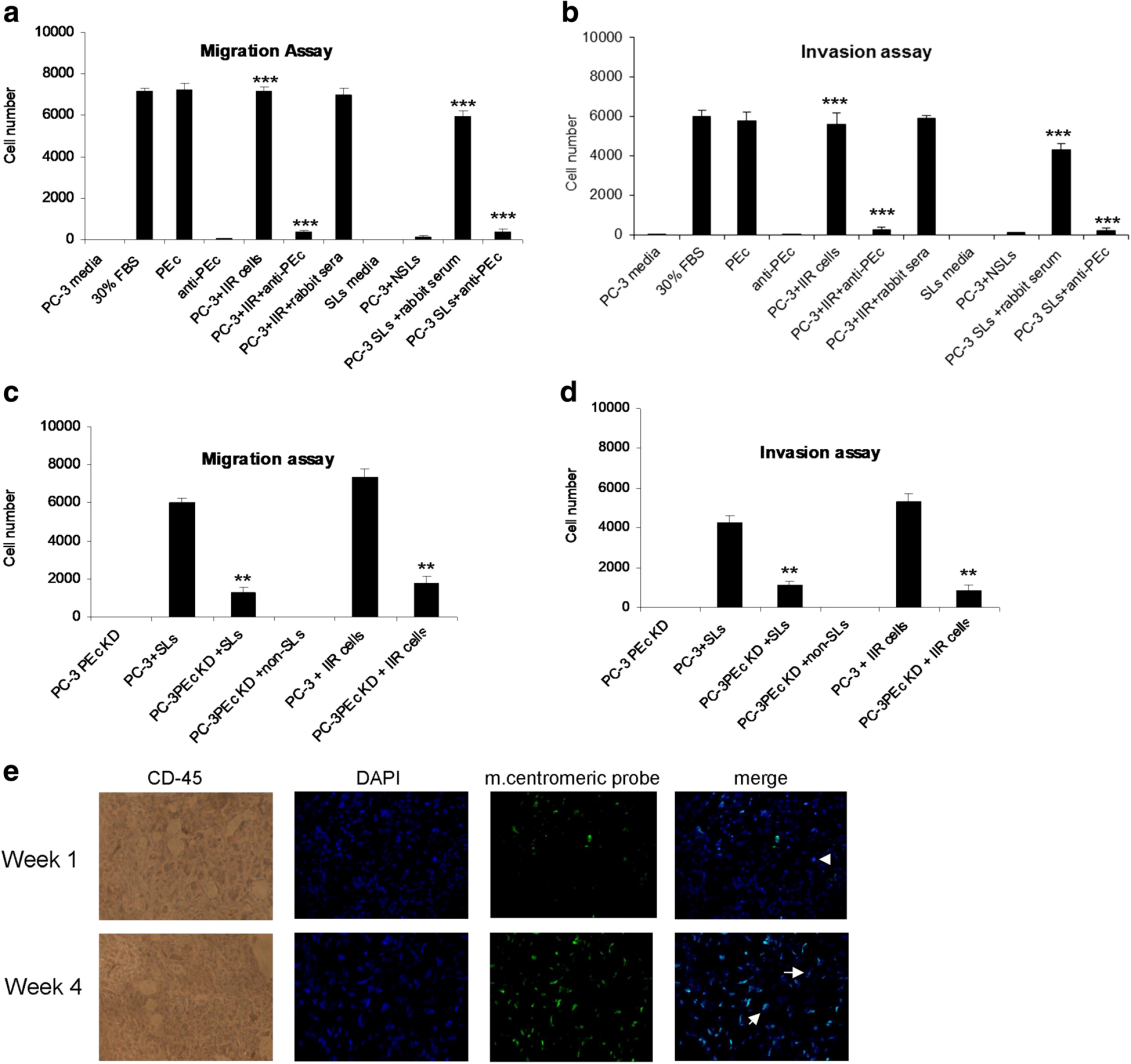
Migration and invasion assays assessing the effects of PEc secreted by the wt and modified PC-3 cells, on MSC mobilization. **a** and **b**: Media obtained from PC-3 cells co-incubated with sensitized lymphocytes or with cells of the human innate IR, induced HMSC migration and invasion. This effect was suppressed by the introduction of the anti-PEc antibody (1/100, 30 min) in both cases for both cell lines. **c** and **d**: The media obtained from PC-3 IGF-1Ec KD cells after co-incubation with sensitized lymphocytes, presented a significantly lower effect on the migration and invasion capacity of the MSC compared to the media obtained from PC-3 cells under the same treatment. Similar results were observed for DU-145 cells. E: Detection of mouse MSC in human tumors in SCID mice as CD-45 negative cells that posses mouse centromeres, in 1 and 4 weeks. The arrows indicate fusion between mouse and human cells (**: *p* < 0.005, ***:*p* < 0.0005)

Serum free media obtained from the co-culturing of PC-3 or DU-145 cells with the SL or with cells of the IIR stimulated the migration and invasion of HMSC in an identical fashion to the effect generated by the synthetic PEc peptide alone. This effect was significantly higher compared to the effect obtained by the media from all the controls (*p* < 0.0001 for every case, *n* = 5, triplicate). This effect was reversed when the media was incubated for 1 h at room temperature with a polyclonal anti-PEc antibody (*p* < 0.0001, *n* = 5, triplicate) (Fig. 2 a, b). Similarly media from prostate cancer cells that had silenced the expression of PEc (PC-3 or DU-145 IGF-1Ec KD cells), collected 48 h after exposure to SL or cells of the human IIR, presented a significantly lower effect on HMSC migration and invasion when compared to the effect caused by the media of PC-3 cells under the same conditions (Student’s t test, *p* = 0.001, *p* = 0.002 respectively, *n* = 5. Error bars refers to s.d) (Fig. 2c, d). This evidence suggests that PEc secretion, induced by the effect of activated lymphocytes or by the innate immunity on prostate cancer cell lines, seems to induce HMSC mobilization. In in vivo conditions tumors from SCID mice were collected at different time intervals and examined simultaneously for the presence of mouse CD-45 and for the existence of mouse centromeric sequences. Mouse mesenchymal cells do not express CD-45.

A number of cells localized in the human tumors collected from SCID mice were observed to express mouse CD-45 and present positive signal for the mouse centromeric sequences. In some of those cells DAPI and FITC (representing the mouse centromeric probe) were exactly aligned, probably representing recruited MSC whereas in other cases FITC was only partially aligned with DAPI suggesting fusion of the mouse MSC with the cells of the human tumor (Fig. 2e).

### Defining the pathway that leads to the IGF-1Ec upregulation

**Fig. 3:**
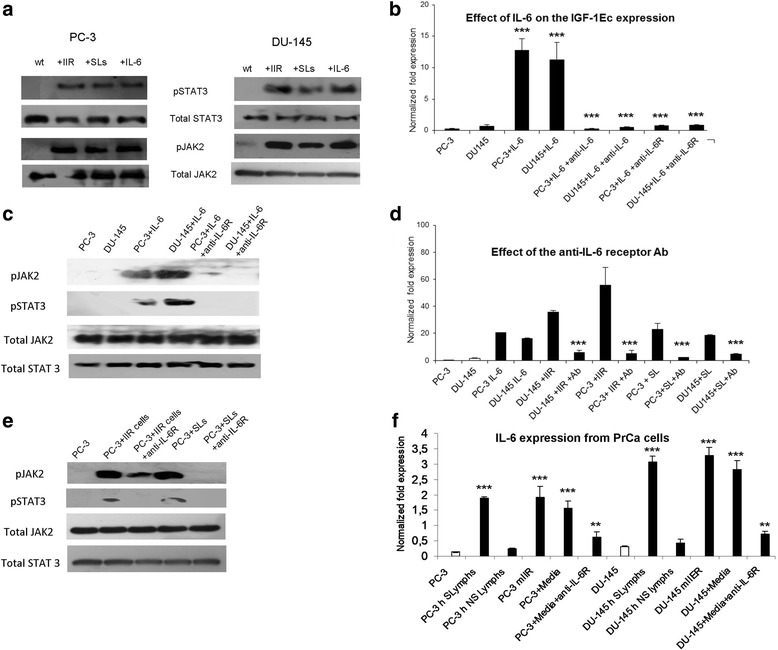
Determination of the effect of IL-6 on the IGF-1Ec expression. **a**: Co-incubation of prostate cancer cell lines with the cells of the innate or adaptive immune system (IIR: innate immune response, SL: sensitized lymphocytes) led to JAK2/STAT3 pathway activation as assessed by western blot analysis. **b**: The exogenous administration of 50 nM of IL-6 for 1 h in both prostate cancer cell lines was associated with a significant increase of IGF-1Ec expression in both cell lines (qRT-PCR). This effect was abolished with the addition of anti-IL-6 or anti-IL-6R antibodies. **c**: Treatment of PC-3 and DU-145 cells with IL-6 led to the induction of JAK2 and STAT3 phosphorylation as assessed by western blot analysis. This effect was reversed when these cell lines were treated with the anti-IL-6R antibody. **d**: In both cell lines the IGF-1Ec upregulation resulted after their exposure to the IR cells (innate or adaptive) was reversed when treated with anti-IL-6R antibody (qRT-PCR). **e**: Effect of the anti-IL-6R antibody on the JAK2 and STAT3 phosphorylation induced by the immune system attack (innate or adaptive) on PC-3 cells (western blot analysis). Antibody treatment led to a significant reduction in the phosporylation status of JAK2 and STAT3. Similar results were observed for the DU-145 cells. F: Both prostate cancer cell lines under the attack of the IR (innate or adaptive) were found to express significant amounts of IL-6 (qRT-PCR) compared to the controls. Conditioned media collected from the co-culturing experiments had the same effect on IL-6 expression when introduced in wt prostate cancer cell lines. The IL-6 upregulation was reversed when the cells were treated with the anti IL-6R antibody (**: *p* < 0.005, ***:*p* < 0.0005)

The IGF-1 gene possesses multiple binding sites for STAT proteins (Varco-Merth & Rotwein, 2014). STAT proteins are activated (phosphorylated) as a response to interleukin receptor (ILR) activation. Interleukins (ILs) are molecules that are involved in the process of the IR and they are secreted by leucocytes. The activation status of all the STAT and JAK proteins was examined by WB analysis. It was found that STAT3 and JAK2 were phosphorylated in both prostate cancer cell lines as a response to immune attack (Fig. 3a).

The JAK2/STAT3 pathway is most commonly activated by the IL-6 receptor. Recent evidence suggests that both prostate cancer cell lines used in this study also possess IL-6 receptor. Treatment of the cancer cells (PC-3 and DU-145) with IL-6 led to significant IGF-1Ec isoform upregulation in both prostate cancer cell lines (*p* = 0.0096 and *p* = 0,001 respectively, students t test, *n* = 5). Blocking of IL-6 using an anti-human IL-6 monoclonal antibody in prostate cancer cell lines treated with IL-6, inhibited the IGF-1Ec production. Similar were the results when an anti-IL-6 receptor antibody used (*p* = 0.001 and *p* = 0,00087 respectively, students t test, *n* = 5) (Fig. 3b).

The treatment of the PC-3and DU-145 cells under the influence of IL-6 with either anti-IL-6 or anti-IL-6R led to inhibition of IGF-1Ec expression. Prior to determine if this effect is associated with the JAK2/STAT3, protein extracts from prostate cancer cells under the influence of IL-6 with and without the treatment of anti-IL-6R antibody were examined for the phosphorylation of JAK2 and STAT3. It was found that treatment of the prostate cancer cell lines with the IL-6 lead to the activation of the JAK2/STAT3 pathway and this activation was abolished when these cells were treated with each of the aforementioned antibody (Fig. 3c).

Prostate cancer cell lines under the influence of the IR (innate or adaptive) presented a significant increase of IGF-1Ec upregulation (similar to the one observed after IL-6 treatment) (*p* < 0.001, students t test, *n* = 3) which was abolished when these cells were treated with anti-IL-6R antibody (Fig. 3d). Furthermore the co-cultures of prostate cancer cell lines with either the cells of the innate or adaptive IR was associated with JAK2/STA3 pathway activation. This effect was abolished when these cells were treated with the anti-IL-6R antibody (Fig. 3e).

Evidence obtained from prostate cancer biopsies suggests that human prostate tumors express IL-6 in many instances (Kall et al., 1976). Due to the fact that mouse IL-6, secreted by mouse macrophages and natural killer cells, is not able to activate the human IL-6R we examined the IL-6 expression in human prostate cancer cells after co-incubation with mouse IIR cells. Both prostate cancer cell lines (PC-3 and DU-145) were examined for IL-6 secretion as a response to immune attack (innate and adaptive) it was found that both cell lines produced significant IL-6 amounts as a response to immune attack after 48 h as assessed by qRT-PCR (*p* = 0.0046 and *p* = 0,0039 respectively students t test, *n* = 3) (Fig. 3f). In order to determine the mechanism that IL-6 secretion is induced from prostate cancer cells (cell to cell activation or IL-6 secreted from activated cells of the immune system) media was collected 12 h after the initiation of co-incubations of the cells of the human IIR with prostate cancer cell lines. It was determined that the media obtained from the co-culturing experiments was able to generate the IL-6 production in prostate cancer cells and this effect was abolished when the anti-IL-6R antibody was introduced into the media (Fig. 3f).

**Fig. 4:**
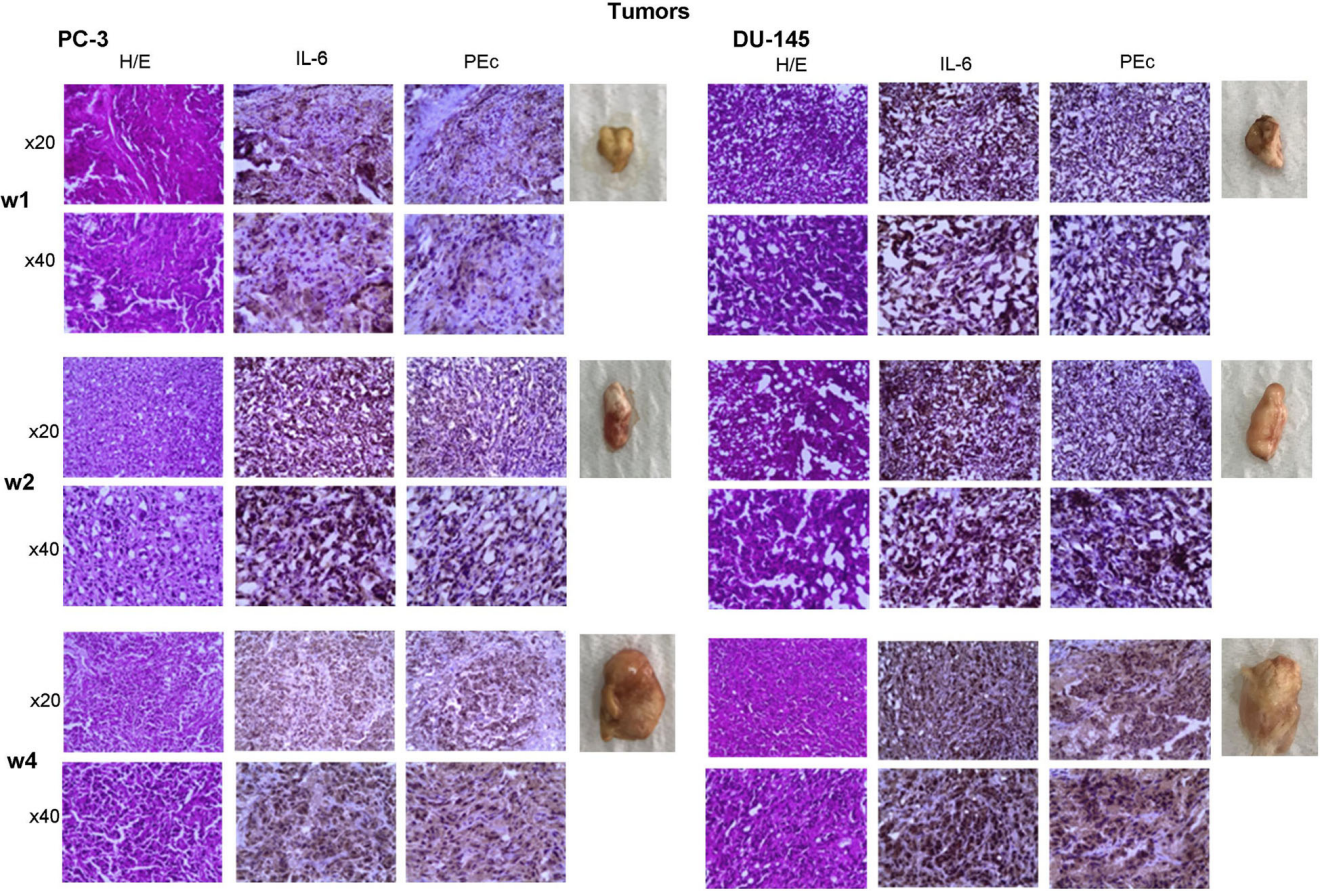
Immunohistochemical detection of IL-6 and PEc in prostate tumors in SCID mice. IL-6 and PEc present proportional expression levels that increase as tumors progress. IL-6 is mainly expressed from cancer cells rather than infiltrating cells of the IR. W1, w2, w4 stands for week 1, 2, 4 after tumor detection by palpitation. (*n* = 3 mice per case were used)

Tumors raised from the inoculation of PC-3 and DU-145 cells in SCID mice were examined for the expression of human IL-6 and PEc at different time intervals. Both cell lines give raise to tumors that expressed IL-6 and PEc and that the expression of both factors co-localises and increases proportionally as the tumors progress (Fig. 4).

## Discussion

Recent evidence supports the oncogenic role of the Ec peptide (PEc) in prostate cancer, where PEc has been associated with the induction of cellular proliferation and metastases (Armakolas et al., 2010; Armakolas et al., 2015). Since, in our previous studies, the PEc was artificially introduced into the cancer cells (exogenously or overexpression models) in this study we examine the condition(s) that prostate cancer cells may secrete PEc physiologically. It was determined that despite the fact that prostate cancer cell lines in in-vitro conditions do not express IGF-1Ec, tumors in SCID mice arising from the same cells do express IGF-1Ec and the IGF-1Ec levels increased as the tumors progressed. Similar seems to be the case in human prostate cancer biopsies where the IGF-1Ec expression levels are significantly associated with tumor stage (the more advanced the tumor the greater the IGF-1Ec expression) (Savvani et al., 2013).

In muscle, IGF-1Ec expression is induced as a response to cellular damage prior to repair (Kandalla et al., 2011). The fact that human tumors are immunogenic (Garg et al., 2015; Galderisi et al., 2010), led to the hypothesis that the immune environment of the host animal may have attacked the xenograft, causing cell damage, leading to PEc secretion. On this ground the effects of the IR (innate and adaptive), in PC-3 and DU-145 cells were investigated, in respect to the IGF-1Ec isoform expression. It was found that both mouse and human cells of the IIR can lead prostate cancer cells to generate IGF-1Ec and to secrete PEc as was determined by in vitro co-culturing experiments. In vivo the activation of the IR was examined by monitoring the MIG-1 levels before and after tumor establishment in SCID mice as well as by detecting the mouse CD-45 expressing cells in the tumors. Similar was the case when the prostate cancer cells were co-incubated with human sensitized lymphocytes. Suggesting that the IR that is raised against prostate cancer may lead to the upregulation of IGF-1Ec isoform and to a further extend to PEc secretion.

Previous studies have implicated the action of PEc, in the repair process of the injured muscle via mobilization of satellite/stem cells (Hill et al., 2003). Recent evidence suggests that cancer cell mediated damage by the IR, mobilises Mesenchymal Stem Cells (MSC) to enter tumor prior to generating repair (Galderisi et al., 2010). This process involves chemokines along with other proteins secreted by cancer cells, which attract HMSCs, and increase their migratory activity (Dwyer et al., 2007; Mishra et al., 2008). In the tumor, MSCs may alter the behaviour of the cancer cells and they may differentiate to carcinoma-associated fibroblasts, which are known to be involved in cancer progression (Lin et al., 2008). Thereby, MSC exhibit tissue repair functions and support angiogenesis which simultaneously contributes to promoting the growth of cancer cells (Karnoub et al., 2007; Mandel et al., 2013). Migration of MSC towards the inflammation site leads to cellular interactions that occur both directly via gap junctions, membrane receptors and nanotubes leading to cell fusion and indirectly via soluble structures and factors (Spaeth et al., 2008).

PEc secreted by prostate cancer cells is capable to mobilize MSC in vitro. In vitro this phenomenon was reversed when the prostate cancer cells under the IR attack were treated with an anti-PEc polyclonal antibody and was abolished when the IGF-1Ec isoform was silenced. In prostate tumors in SCID mice we detected a number of mouse CD-45 negative cells in the tumor and in some instances we also observed fusion of nuclei suggesting repair.

**Fig. 5:**
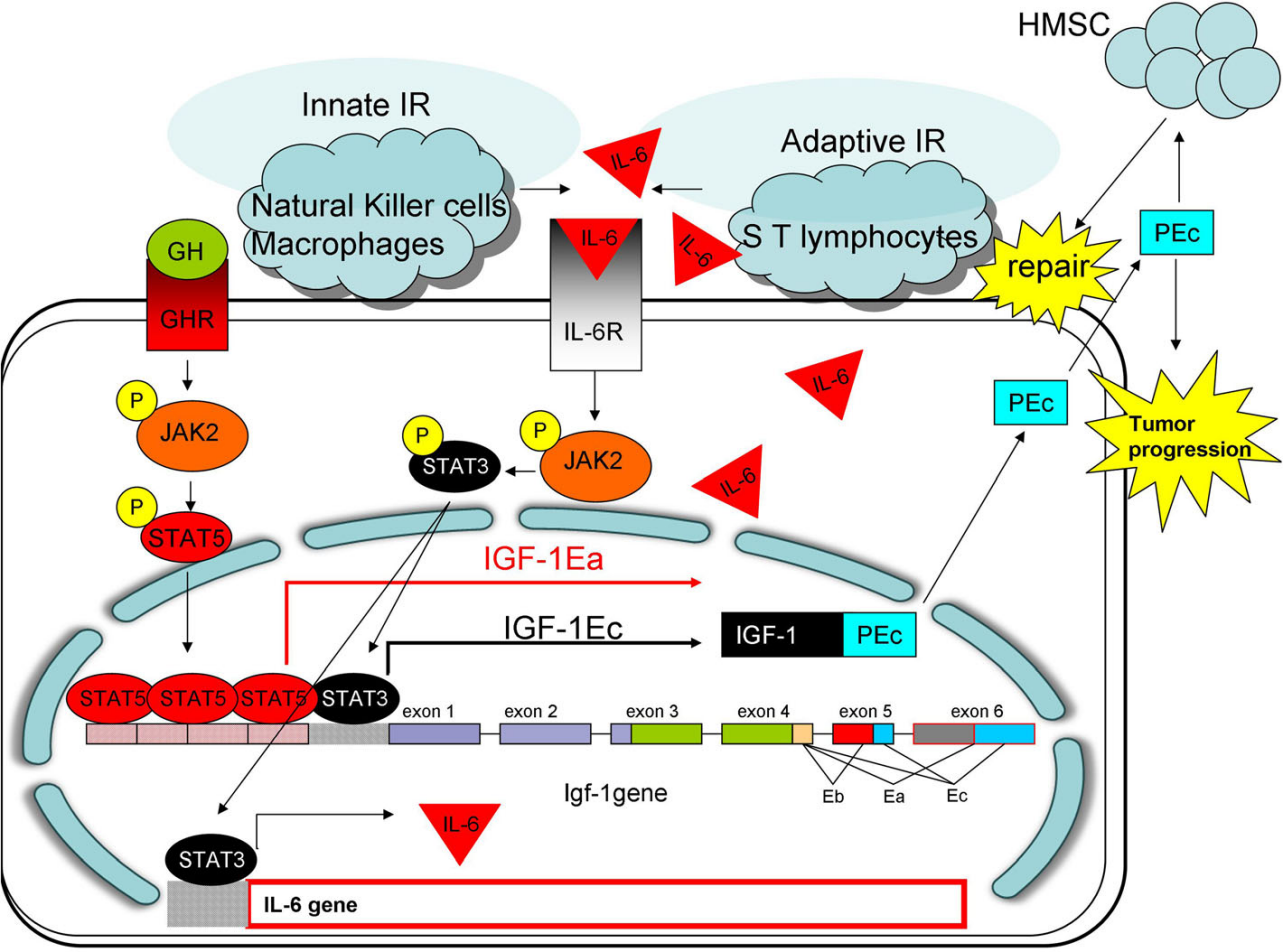
The effect of the IR on the expression of the IGF-1Ec isoform. GH binding to GHR leads to IGF-1Ea upregulation through JAK2/STA5 pathway. In a similar manner, IL-6 is initially produced by the cells of the immune system attacking prostate tumor.. Binding of IL-6 on IL-6R leads to the upregulation of the IGF-1Ec isoform and to the secretion of PEc as well as to IL-6 secretion from prostate cancer cells, by activating the JAK2/STAT3 pathway. PEc secretion leads to tumor progression and metastases acting in an autocrine and/or paracrine manner and it mobilizes MSC towards the tumor, prior to repair

The question raised at this point was how the IR leads to the IGF-1Ec upregulation. Previous studies suggest that the igf-1 gene possesses bSTAT response elements in its structure. Growth Hormone-activated (GH) STAT5B promotes transcription of the IGF-I gene (Fig. 5). STAT1 and STAT3 present a weaker profile of in vitro binding to STAT DNA elements compared to STAT5B in the igf-1 gene, and are less potent inducers of gene transcription (Haque & Sharma, 2006).

The major effectors of the STAT system are cytokines. Cytokine signals are, in general, transient in nature. Therefore, under normal physiological conditions, initiation and attenuation of cytokine signals are tightly controlled via multiple cellular and molecular mechanisms. Aberrant activation of cytokine signalling pathways is, however, found under a variety of patho-physiological conditions including prostate cancer (Haque & Sharma, 2006; Villarino et al., 2015).

Having that in mind, the phosphorylation of the 6 STAT and of 3 JAK proteins that are activated as a response to immune attack was examined. It was determined that the prostate cancer cells treated with the IIR cells or sensitized lymphocytes presented JAK2 and STAT3 phosphorylation. This pathway is activated by IL-6.

Normally IL-6R is expressed only by hepatocytes, neutrophils, monocytes/macrophages and some lymphocytes (Azevedo et al., 2011). Recent evidence suggests that IL-6 may have a crucial role in prostate cancer progression through autocrine on tumor cells or paracrine activity on normal cells in the tumor microenvironment. It has been found that prostate tumor cells produce large amounts of IL-6 and express its receptors, IL-6R (gp80) and gp130, allowing them to respond in an autocrine manner to IL-6 (Mishra et al., 2008; Jones et al., 2001). Both prostate cancer cell lines used in this study possess the IL-6 receptor. Although the exact effects or the mechanisms that IL-6 is involved on prostate cancer cells is not yet known, it has been suggested that IL-6 can modulate the metastatic process as well as the transition from hormone-dependent prostate cancer to castration resistance prostate cancer (Nguyen et al., 2014).

Prostate cancer cells used in this study expressed IL-6 as a response to immune attack that seemed to be associated with the IGF-1Ec upregulation and PEc secretion, through the activation of JAK2/STA3 pathway (Fig. 5). IL-6 expression in cancer cells seems to be initiated by IL-6 produced by the cells of the IR that through IL-6R leads to the production of IGF-1Ec isoform and to the expression of IL-6 through its canonical pathway. The tumor IL-6 is then secreted and generates its positive feedback in an autocrine fashion.

The correlation of IL-6 and PEc is fortified by the fact that both these factors possess similar oncogenic role that is associated with tumor progression and metastases. The fact that the blockade of IL-6 or of the IL-6R lead to PEc downregulation together with the evidence obtained by IHC which indicates that the expression of both of these factors: a) is co-localized in the tumor, b) is proportional and c) it increases as the tumors progress, may be indicative that the oncogenic effect of IL-6 such as the involvement in EMT may be generated through IGF-1Ec isoform and PEc secretion.

## Conclusion

Exogenous IL-6 leads to the production and secretion of IL-6 and PEc from prostate cancer cells and tumors, by activating IL-6R and JAK2/STAT3 pathway. PEc secretion from the tumors, apart from its oncogenic properties, is also associated with the MSC mobilisation and tumor repair. On the other hand IL-6 secretion from the tumor leads to its positive feedback and to the continuous secretion of PEc.

## Additional files

Additional file 1: Human Lymphocyte isolation and characterization. Human lymphocytes were isolated from blood and characterized by flow cytometry using the surface markers CD-45 and CD-3 for T lymphocytes (76.75% of the cells) and CD-45 and CD-20 for B lymphocytes (14.18%). Lymphocytes accounted for the 90.93% of the total number of cells isolated. (JPEG 234 kb)

Additional file 2: Characterisation of primary human mesenchymal cells. E-cadherin and Vimentin expression as assessed by immunofluorescence staining. The primary isolated human mesenchymal cells expressed Vimentin and they did not express E-cadherin. As a positive control for E-cadherin staining and negative control for Vimentin staining we used the wtPC-3 cells (prostate cancer cells of epithelial origin). (JPEG 184 kb)

Additional file 3: Verification of the immune attack on prostate cancer cells. A: Determination of mouse MIG-1 expression using qRT-PCR. Mouse MIG-1 mRNA expression was significantly increased after 6 and 12 hours co-incubation of the cells of the innate immune response (IIR) with wtPC-3 cells as compared to the negative controls (*p* < 0.001 for both time intervals. As a positive control we used SCID mouse white blood cells incubated with IFN γ (20 Units) for 6 hours. (Student’s *t* test, *P* < 0.001, triplicate, error bars refer to s.d ). Lane 1: wtPC-3 cells, 2: SCID mouse white blood cells at 0 hrs and 3: at 6 hours, in tissue culture conditions, 4: MIG-1 expression in SCID mouse blood cells after 6 hours incubation with 20 Units of IFNγ. (Student’s *t* test, *p* < 0.01, triplicate. Error bars refers to s.d). B: Determination of the viable PC-3 or DU-145 cells after co-incubation with cells of the human IIR or with human sensitized lymphocytes (SL), for 48 hours (Trypan blue exclusion assay). Prostate cancer cells presented a significant decrease in every case. (Student’s *t* test, *p* < 0.008, triplicate. Error bars refers to s.d). (NSL: Non-sensitized lymphocytes, IIR: Innate Immune Response, SL: Sensitized Lymphocytes. (JPEG 255 kb)

Additional file 4: Effect of the immune response on IGF-1Eb expression. A and B the human innate immune response is associated with significant IGF-1Eb upregulation in prostate cancer cell lines. C, D similar was the case with the human adaptive immune response. E exogenous administration of PEc on prostate cancer cells and PEc overexpression models suggest that IGF-1Eb uprgulation does or does not depend on PEc. (JPEG 157 kb)
