# Supplementary figures and images for: IL-6 is associated to IGF-1Ec upregulation and Ec peptide secretion, from prostate tumors
Source: Mol Med. 2018 Mar 15;24:6. doi: 10.1186/s10020-018-0003-z (PMC6016866; doi:10.1186/s10020-018-0003-z)

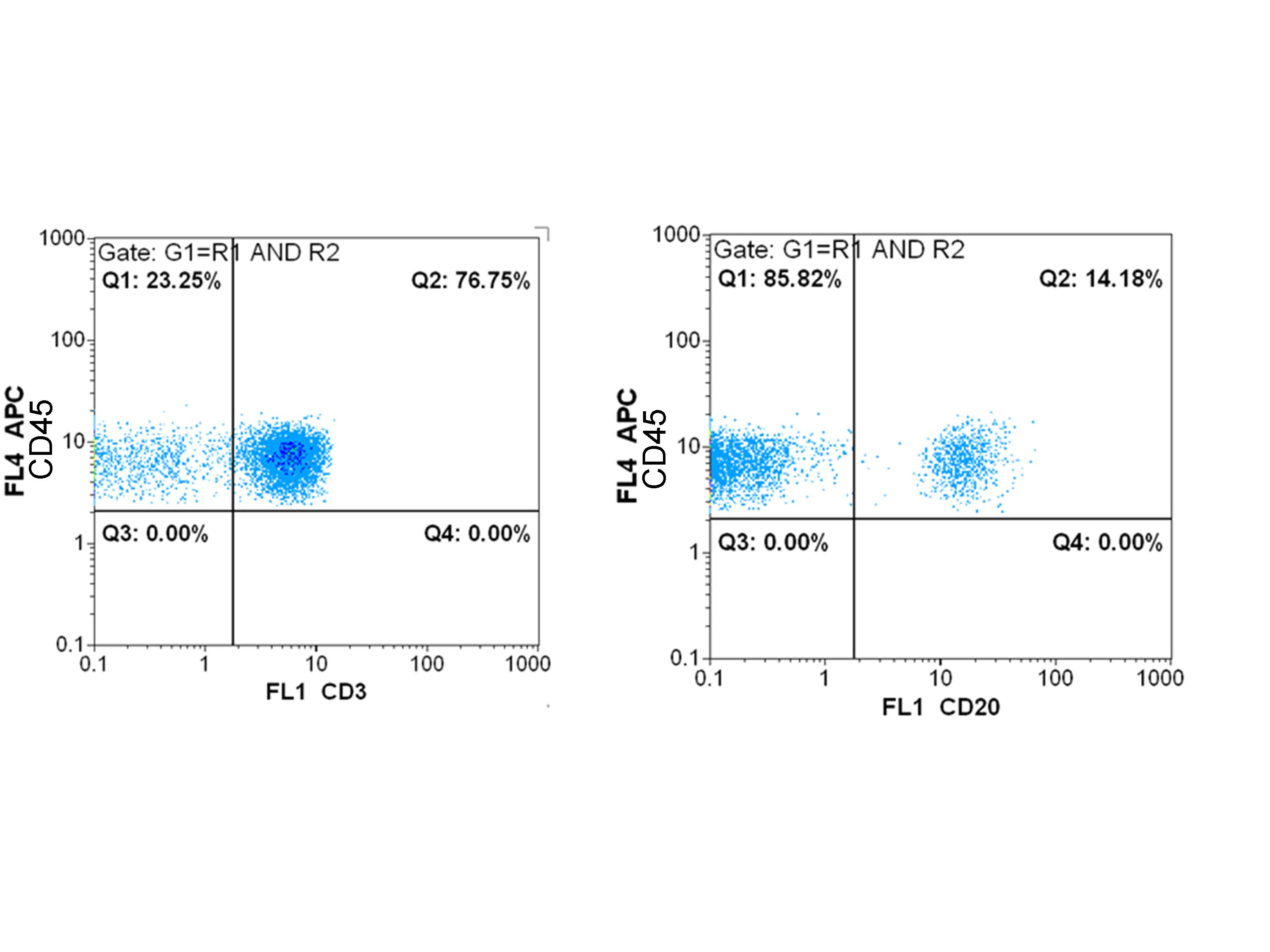

Supplement: Supplementary file 1 — Human Lymphocyte isolation and characterization. Human lymphocytes were isolated from blood and characterized by flow cytometry using the surface markers CD-45 and CD-3 for T lymphocytes (76.75% of the cells) and CD-45 and CD-20 for B lymphocytes (14.18%). Lymphocytes accounted for the 90.93% of the total number of cells isolated. (JPEG 234 kb) [file 10020_2018_3_MOESM1_ESM.jpg]

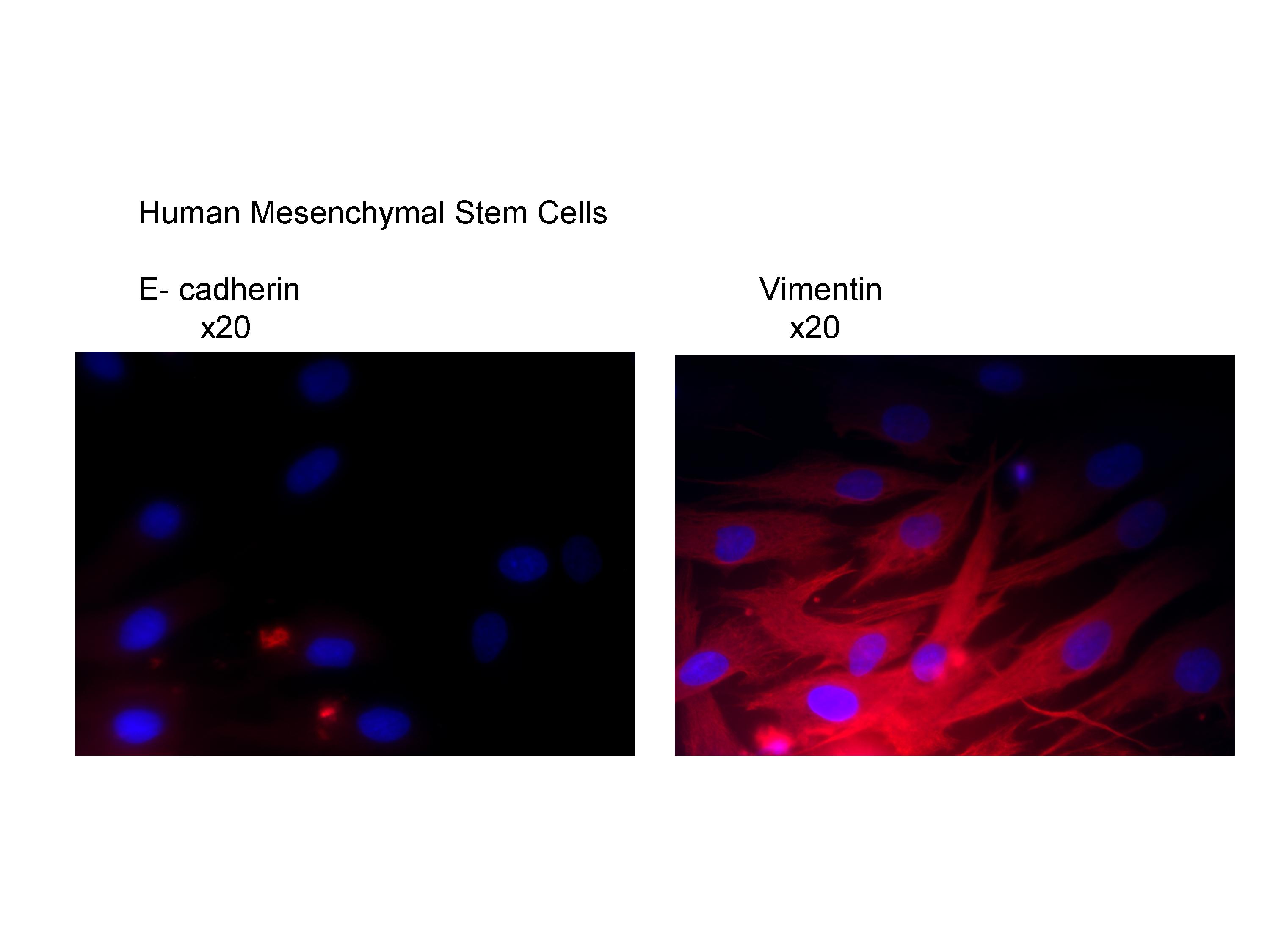

Supplement: Supplementary file 2 — Characterisation of primary human mesenchymal cells. E-cadherin and Vimentin expression as assessed by immunofluorescence staining. The primary isolated human mesenchymal cells expressed Vimentin and they did not express E-cadherin. As a positive control for E-cadherin staining and negative control for Vimentin staining we used the wtPC-3 cells (prostate cancer cells of epithelial origin). (JPEG 184 kb) [file 10020_2018_3_MOESM2_ESM.jpg]

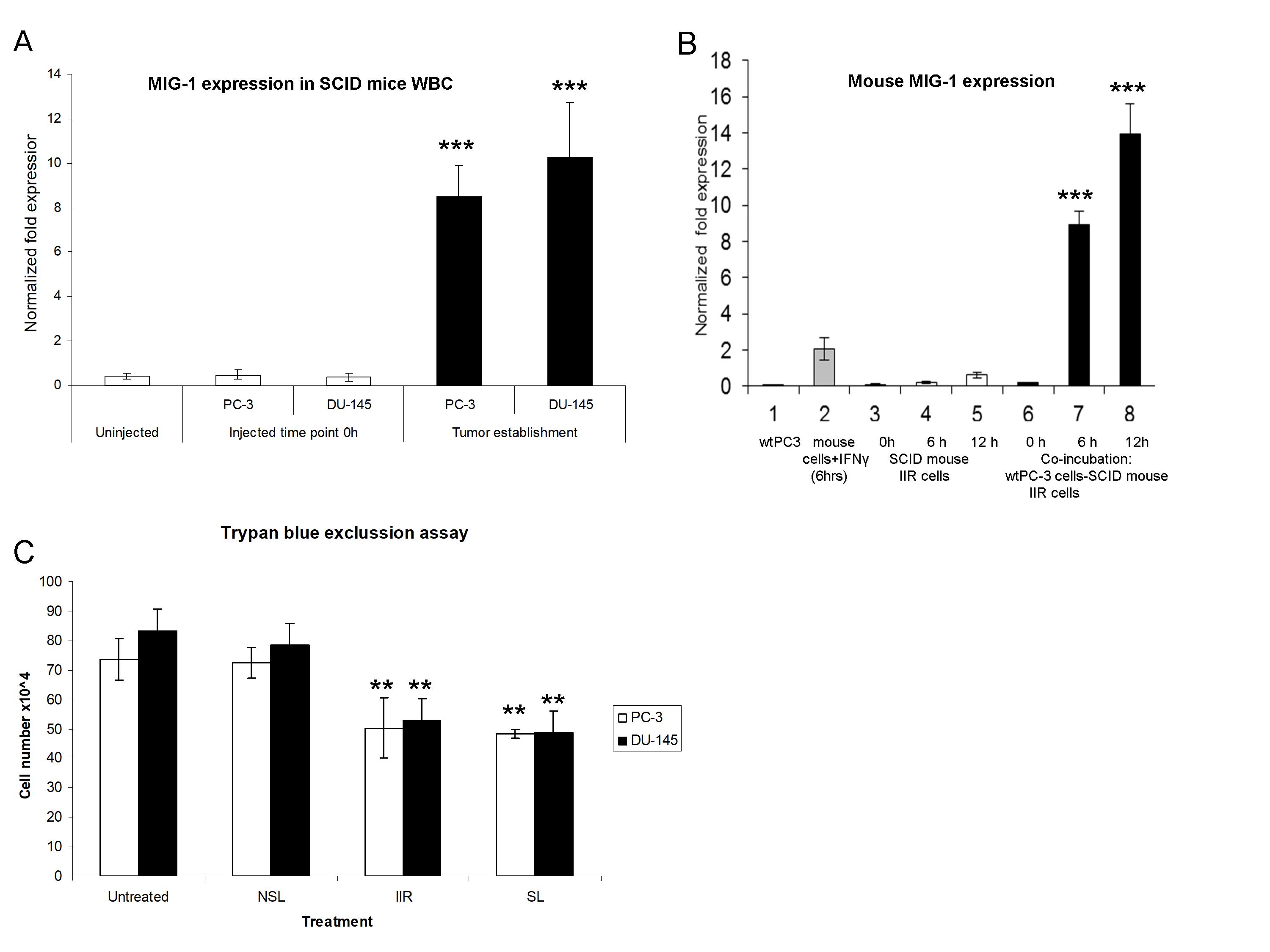

Supplement: Supplementary file 3 — Verification of the immune attack on prostate cancer cells. A: Determination of mouse MIG-1 expression using qRT-PCR. Mouse MIG-1 mRNA expression was significantly increased after 6 and 12 hours co-incubation of the cells of the innate immune response (IIR) with wtPC-3 cells as compared to the negative controls (p < 0.001 for both time intervals. As a positive control we used SCID mouse white blood cells incubated with IFN γ (20 Units) for 6 hours. (Student’s t test, P < 0.001, triplicate, error bars refer to s.d ). Lane 1: wtPC-3 cells, 2: SCID mouse white blood cells at 0 hrs and 3: at 6 hours, in tissue culture conditions, 4: MIG-1 expression in SCID mouse blood cells after 6 hours incubation with 20 Units of IFNγ. (Student’s t test, p < 0.01, triplicate. Error bars refers to s.d). B: Determination of the viable PC-3 or DU-145 cells after co-incubation with cells of the human IIR or with human sensitized lymphocytes (SL), for 48 hours (Trypan blue exclusion assay). Prostate cancer cells presented a significant decrease in every case. (Student’s t test, p < 0.008, triplicate. Error bars refers to s.d). (NSL: Non-sensitized lymphocytes, IIR: Innate Immune Response, SL: Sensitized Lymphocytes. (JPEG 255 kb) [file 10020_2018_3_MOESM3_ESM.jpg]

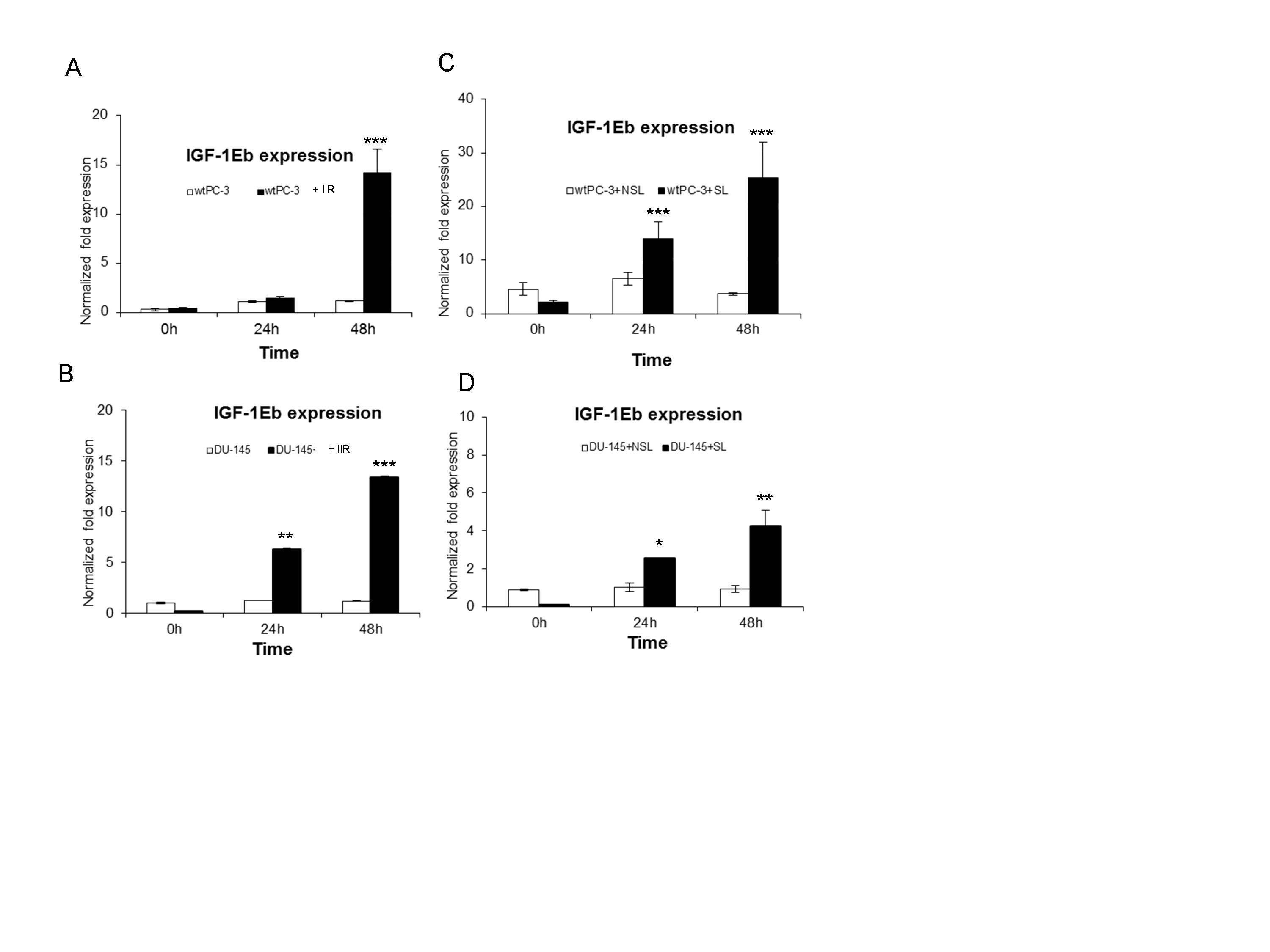

Supplement: Supplementary file 4 — Effect of the immune response on IGF-1Eb expression. A and B the human innate immune response is associated with significant IGF-1Eb upregulation in prostate cancer cell lines. C, D similar was the case with the human adaptive immune response. E exogenous administration of PEc on prostate cancer cells and PEc overexpression models suggest that IGF-1Eb uprgulation does or does not depend on PEc. (JPEG 157 kb) [file 10020_2018_3_MOESM4_ESM.jpg]
